# Ascending Vaginal Infection Using Bioluminescent Bacteria Evokes Intrauterine Inflammation, Preterm Birth, and Neonatal Brain Injury in Pregnant Mice

**DOI:** 10.1016/j.ajpath.2018.06.016

**Published:** 2018-10

**Authors:** Natalie Suff, Rajvinder Karda, Juan A. Diaz, Joanne Ng, Julien Baruteau, Dany Perocheau, Mark Tangney, Peter W. Taylor, Donald Peebles, Suzanne M.K. Buckley, Simon N. Waddington

**Affiliations:** ∗ Gene Transfer Technology Group, University College London, London, United Kingdom; † Preterm Birth Group, Department of Maternal and Fetal Medicine, Institute for Women's Health, University College London, London, United Kingdom; ¶ School of Pharmacy, University College London, London, United Kingdom; ‡ Department of Metabolic Medicine, Great Ormond Street Hospital for Children NHS Foundation Trust, London, United Kingdom; § SynBio Centre, University College Cork, Cork, Ireland; ‖ MRC Antiviral Gene Therapy Research Unit, Faculty of Health Sciences, University of the Witwatersrand, Johannesburg, South Africa

## Abstract

Preterm birth is a serious global health problem and the leading cause of infant death before 5 years of age. At least 40% of cases are associated with infection. The most common way for pathogens to access the uterine cavity is by ascending from the vagina. Bioluminescent pathogens have revolutionized the understanding of infectious diseases. We hypothesized that bioluminescent *Escherichia coli* can be used to track and monitor ascending vaginal infections. Two bioluminescent strains were studied: *E. coli* K12 MG1655-*lux*, a nonpathogenic laboratory strain, and *E. coli* K1 A192PP-*lux2*, a pathogenic strain capable of causing neonatal meningitis and sepsis in neonatal rats. On embryonic day 16, mice received intravaginal *E. coli* K12, *E. coli* K1, or phosphate-buffered saline followed by whole-body bioluminescent imaging. In both cases, intravaginal delivery of *E. coli* K12 or *E. coli* K1 led to bacterial ascension into the uterine cavity, but only *E. coli* K1 induced preterm parturition. Intravaginal administration of *E. coli* K1 significantly reduced the proportion of pups born alive compared with *E. coli* K12 and phosphate-buffered saline controls. However, in both groups of viable pups born after bacterial inoculation, there was evidence of comparable brain inflammation by postnatal day 6. This study ascribes specific mechanisms by which exposure to intrauterine bacteria leads to premature delivery and neurologic inflammation in neonates.

Preterm birth is a serious obstetric and global health problem. It is defined as delivery before 37 weeks' gestation and it is known to affect approximately 11% of pregnancies worldwide.^1^ It is the leading cause of death in infants younger than 5 years of age and it is associated with serious morbidity in the surviving infants.^2^, ^3^ The rates of preterm birth have remained stable over the years and this is largely because of a lack of understanding of the mechanisms behind preterm birth as well as the paucity of effective preventive treatments. Improving our understanding may help the development of novel therapies.

Infection and inflammation have been linked commonly to preterm birth and are estimated to be associated with up to 40% of preterm deliveries.^4^ In clinical studies, the presence of certain intrauterine bacteria is associated with preterm prelabor rupture of membranes and spontaneous preterm birth.^5^ Evidence from animal models also has confirmed this link by showing that inoculation of the intrauterine cavity with live bacteria, or bacterial toxins such as lipopolysaccharide (LPS), can lead to preterm birth.^6^, ^7^, ^8^ Ascending vaginal infection is thought to be the most common route by which bacteria gain access into the uterine cavity.^9^ The main supporting evidence for this is based on the association between the bacterial species identified in the fetal membranes and placenta and those normally found in the lower genital tract.^5^

Animal models of infection-related preterm birth provide useful insight into the mechanisms that regulate infection, inflammation, and preterm parturition. Animal models of ascending vaginal infection are important in providing mechanistic data on infection-related preterm birth and in validating novel preventative treatments that could be clinically translatable. Murine models of intravaginal group B *Streptococcus*, *Escherichia coli*, and *Ureaplasma urealyticum* ascending infection have recapitulated the process of ascending vaginal infection.^8^, ^10^, ^11^, ^12^ They have provided insight into the pathogenesis of preterm birth, such as the protective role of cervical hyaluronan in preventing ascending infection.^8^

There now are several studies that support an association between intrauterine inflammation, preterm birth, and cerebral palsy.^13^, ^14^, ^15^ The relative contribution of prematurity and inflammation to preterm brain injury has yet to be fully elucidated. In the long-term follow-up data from the Overview of the Role of Antibiotics in Curtailing Labor and Early Delivery (ORACLE) II trial, there was a higher incidence of cerebral palsy in children whose mothers had received antibiotics after spontaneous preterm birth.^16^ This could suggest that antibiotic use in preterm birth may help to delay delivery, but may lead to prolonged exposure of the fetal brain to a detrimental intrauterine inflammatory environment. Rodent studies have shown that low-dose LPS, insufficient to cause premature delivery, still can lead to significant brain injury.^17^ Preterm birth often is associated with bacteria of low pathogenicity, with the *Ureaplasma* species being the most common bacteria isolated from the amniotic cavity of patients with preterm chorioamnionitis.^18^

The use of engineered bioluminescent pathogens to model infectious processes has become increasingly common. The bacterial *lux* operon encodes enzymes that are involved in a light-emitting reaction; this is catalyzed by a bacterial luciferase that is encoded by the *luxA* and *luxB* genes and a multienzyme complex (encoded by the *luxC*, *luxD*, and *luxE* genes), which is responsible for the regeneration of the aldehyde substrate from the fatty acid produced in the initial luciferase reaction. The main advantage of the lux operon system is the ability to express the enzymes that synthesize the substrate, rendering addition of an exogenous substrate unnecessary.^19^ The lux operon from bacteria such as *Photorhabdus luminescens* have been used to genetically modify other bacteria to confer them with bioluminescence.

Here, we show that two bioluminescent strains of *E. coli*, *E. coli* K12 MG1655-*lux* (*E. coli* K12) and *E. coli* K1 A192PP-*lux2* (*E. coli* K1), can cause ascending infection in pregnant mice. *E. coli* K12 is a noninvasive, nonpathogenic strain of *E. coli* commonly used in molecular biology.^20^, ^21^ *E. coli* K1 is a pathogenic strain of *E. coli* that, similar to group B *streptococcus*, is responsible for causing neonatal meningitis and sepsis in humans by vertical transmission from the mother. Using these different strains of bioluminescent *E. coli*, we have explored the mechanisms of preterm parturition, as well as the effects of exposure to intrauterine inflammation on neonatal brain development.

## Materials and Methods

### Animals and Treatments

All animal studies were conducted under UK Home Office license 70/8030 and were approved by the University College London ethical review committee.

C57BL/6 Tyr^c-2J^ mice were obtained from the Jackson laboratory (Bar Harbor, ME) and adult mice (age, 6 to 12 wk) were time mated. The following morning (when a vaginal plug was noted) was designated as embryonic day 0. The ascending vaginal infection model was developed using *E. coli* K12 MG1655-*lux*^22^ and *E. coli* K1 A192PP-*lux2*^23^ modified to contain the *lux* operon from *P. luminescens*. Twenty microliters of midlogarithmic-phase *E. coli* (1 × 10^9^ *E. coli* K12 or 1 × 10^2^ *E. coli* K1 resuspended in 10 mmol/L phosphate buffer) or phosphate-buffered saline (PBS) was delivered into the vagina of mice anesthetized with isoflurane using a 200-mL pipette tip. This study used the *E. coli* 018:K1 A192PP strain with the *luxCDABE* transposon integrated by mini-Tn5 mutagenesis.^23^ After bacterial administration, mice were placed in individual cages and monitored continuously with individual closed-circuit television cameras and a digital video recorder. The time to delivery was recorded and defined as the number of hours from the time of bacterial administration to delivery of the first pup. The number of live and dead pups was recorded. Living pups were weighed daily and were sacrificed if a 10% daily increase in body weight was not maintained.

### Whole-Body Bioluminescence Imaging

Adult mice were anesthetized with isoflurane (Abbott Laboratories, Lake Bluff, IL). Neonatal mice (up to postnatal day 6) remained conscious during imaging.^24^ Mice were imaged using a cooled charged-coupled device camera (IVIS Machine; Perkin Elmer, Coventry, UK) for between 1 second and 5 minutes. The regions of interest were measured using Living Image software version 4.5 (Perkin Elmer) and expressed as photons per second per centimeter squared per steradian.

### Tissue Collection

For tissue collection, mice were anesthetized using isoflurane. The right atrium was incised and PBS was injected into the left ventricle. Pregnant mice were sacrificed 18 hours after intravaginal infection. Placental tissue was stored in 4% paraformaldehyde. Embryos were stored in 10% neutral-buffered formalin. In a separate cohort of pregnant mice, the uterus, placenta, and fetal membranes were collected and stored in RNAlater (Thermo Fisher Scientific, Paisley, UK) at −80°C for quantitative PCR analysis. Separate cohorts of neonatal pups were sacrificed at postnatal day 6. The brains were either stored at −20°C for protein analysis or fixed in 4% paraformaldehyde.

### RNA Synthesis, cDNA Synthesis, and Quantitative PCR

Total RNA was extracted from the uterus, fetal membranes, and placental tissue, which was collected 18 hours after intravaginal infection using the RNeasy mini kit (Qiagen, Manchester, UK), as per the manufacturer's guidelines. Total RNA was reverse-transcribed with the High Capacity cDNA Reverse Transcription kit (Applied Biosystems, Cheshire, UK). Primer sets were obtained from Life Technologies (Paisley, UK) and quantitative PCR was performed in the presence of SYBR green (Applied Biosystems) (Table 1). Target gene expression was normalized for RNA loading by using *GAPDH*, and the expression in each sample was calculated relative to a calibrator sample (uninfected D17 uterus, fetal membranes, or placenta), using the 2^−ΔΔCt^ method of analysis.^25^ All quantitative PCR analyses were performed on an Applied Biosystems QuantStudio 3 instrument (Applied Biosystems).

**Table 1:** Quantitative PCR Primer Sets

Primer	Sequence
*Gapdh*	
F	5′-ACTCCACTCACGGCAAATTC-3′
R	5′-TCTCCATGGTGGTGAAGACA-3′
*Il1b*	
F	5′-CAGGCAGGCAGTATCACTCA-3′
R	5′-AGCTCATATGGGTCCGACAG-3′
*Tnfa*	
F	5′-TATGGCTCAGGGTCCAACTC-3′
R	5′-CTCCCTTTGCAGAACTCAGG-3′
*Il6*	
F	5′-AGTTGCCTTCTTGGGACTGA-3′
R	5′-TCCACGATTTCCCAGAGAAC-3′
*Cxcl-1*	
F	5′-GCCTATCGCCAATGAGCTG-3′
R	5′-AAGGGAGCTTCAGGGTCAAG-3′
*Cxcl2*	
F	5′-CAGTGCCTCCAACAAGCTTC-3′
R	5′-CATTGACAGCGCAGTTCACT-3′
*Cxcr2*	
F	5′-TTCTGCTACGGGTTCACACT-3′
R	5′-TTAAGGCAGCTGTGGAGGAA-3′

F, forward; R, reverse.

### Immunoperoxidase Immunohistochemistry

Individual brain sections were mounted and dried on chrome gelatin-coated Superfrost-plus slides (VWR, Lutterworth, UK). To visualize CD68 immunoreactivity, sections were treated with 30% H_2_O_2_ in Tris-buffered saline (TBS) for 30 minutes. They were blocked with 15% rabbit serum in TBS-Tween 20 for 30 minutes. This was followed by the addition of primary antibody, rat anti-mouse CD68 (1:100; Bio-Rad, Watford, UK) in 10% serum, and TBS-Tween 20 and left overnight at 4°C. The following day the sections were treated with secondary biotinylated rabbit anti-mouse antibody (1:1000; Vector Laboratories, Burlingame, CA) and goat anti-rat (1:1000 dilution; Vector Laboratories) in 10% serum in TBS-Tween 20 for 2 hours. The sections were incubated for a further 2 hours with Vectastain ABC solution (Vector Laboratories). A total of 0.05% of 3,3′-diaminobenzidine was added and left for 2 minutes. Sections were transferred to ice-cold TBS. The slides were dehydrated in 100% ethanol and placed in Histoclear (National Diagnostics, Nottingham, UK) for 30 minutes before adding a coverslip with DPX mounting medium (Sigma-Aldrich, Dorset, UK).

### Terminal Deoxynucleotidyl Transferase-Mediated dUTP Nick-End Labeling Assay

Brain sections were mounted on chrome gelatin-coated Superfrost-plus slides and dried overnight before fixation in 4% paraformaldehyde. Slides were transferred to a methanol and 10% H_2_O_2_ solution for 15 minutes at room temperature. Slides were transferred to 0.1 mol/L phosphate buffer solution. Terminal deoxynucleotidyl transferase-mediated dUTP nick-end labeling (TUNEL) staining solution (13 μL terminal deoxynucleotidyl transferase, 19.5 μL biotinylated deoxyuridine triphosphate, 1.3 mL cacodylate buffer, and 11.67 mL dH_2_O) was prepared on ice and slides were incubated for 2 hours. TUNEL stop solution (300 mmol/L sodium chloride, 30 mmol/L sodium citrate) was prepared and applied for 10 minutes. Sections were incubated for 1 hour with Vectastain ABC solution. A solution (3,3′-diaminobenzidine cobalt-nickel) was added and left for approximately 5 minutes. The sections were left to dry and then were dehydrated for 10 minutes in 100% ethanol and placed in Histoclear for 30 minutes before adding a coverslip with DPX mounting medium.

### Fluorescence Immunohistochemistry

Paraffin-embedded slides were dewaxed in Histoclear and rehydrated in ethanol. H_2_O_2_ treatment was omitted. To block nonspecific binding, slides were incubated in 15% goat serum for 30 minutes. Rabbit anti–*E. coli* primary antibody (1:1000; Abcam, Cambridge, UK) was added and slides were left overnight at 4°C. Sections were incubated for 2 hours with a fluorescent goat anti-rabbit secondary antibody (1:1000, Alexa Fluor 488; Invitrogen, Carlsbad, CA). The sections were treated with DAPI (5 mg/mL) in the dark for 2 minutes and were transferred to ice-cold TBS. The slides then were left to dry followed by application of a coverslip with Fluromount G (Southern Biotech, Birmingham, AL).

### Cytokine Enzyme-Linked Immunosorbent Assays

Tissue lysates were prepared by homogenization in protein lysis buffer. Total protein concentration was quantified using the Pierce BCA Protein Assay kit (Thermo Fisher Scientific, Glasgow, UK) as per the manufacturer's guidelines. Enzyme-linked immunosorbent assay kits for mouse IL-1β, tumor necrosis factor-α, and IL-6 (R&D Systems, Minneapolis, MN) were used to quantify cytokine levels according to the manufacturer's guidelines.

### Statistics

Data are expressed as means ± SEM. Time-to-delivery data were log-transformed before analysis, and the proportion of live born pups was arc-sin transformed before analysis. Data were analyzed by unpaired *t*-tests and one-way analysis of variance (with *post hoc* Bonferroni tests). All statistical analyses were performed with GraphPad Prism software version 7.0 (GraphPad Software, La Jolla, CA). *P* < 0.05 was considered statistically significant.

## Results

### Ascending Vaginal Infection and Preterm Birth Using Bioluminescent Strains of *E. coli*

**Figure 1:**
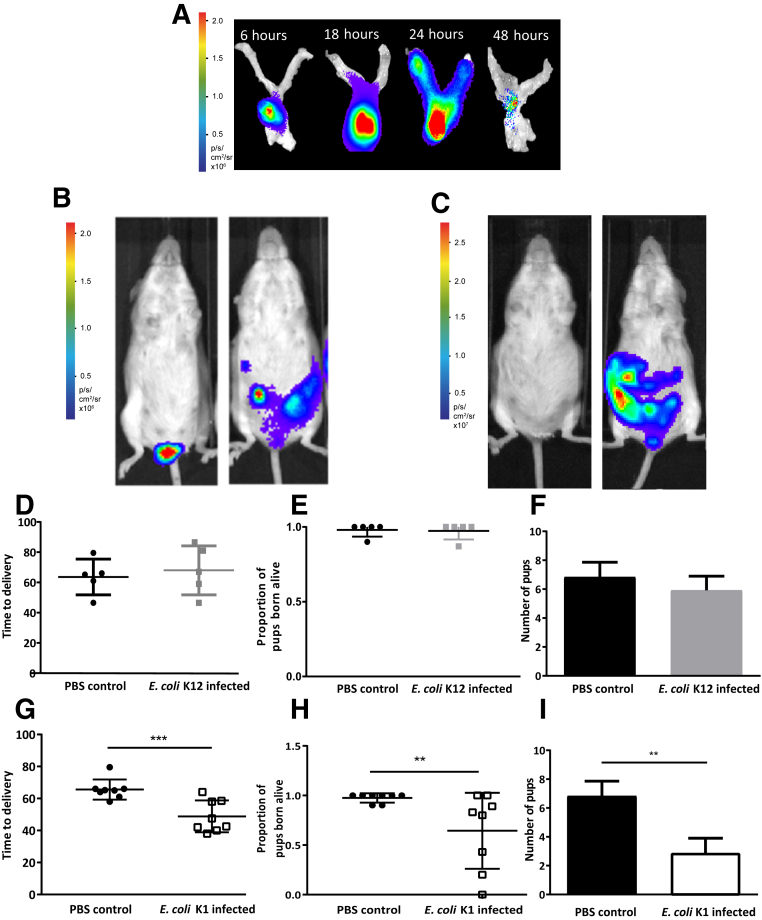
Nonpathogenic *Escherichia coli* K12 and pathogenic *E. coli* K1 can ascend into the pregnant uterine cavity, leading to premature delivery in the *E. coli* K1 group. **A:** Nonpregnant mice received intravaginal *E. coli* K12 bacteria and the time course of bacterial ascent was visualized in the nonpregnant reproductive tract. Bacteria traversed the cervix and ascended into the uterine horns by 18 hours and reached the top of the uterine horns by 24 hours. **B:** In pregnant mice, *E. coli* K12 bacteria ascended into the pregnant uterine cavity by 24 hours. **C:** After *E. coli* K1 administration, bacteria ascended into the pregnant uterine cavity over 24 hours. **D–F:** Time to delivery (**D**), the proportion of pups born alive (**E**), and litter size (**F**) were determined in dams who received intravaginal phosphate-buffered saline (PBS) or intravaginal *E. coli* K12. **G–I:** Time to delivery (**G**), the proportion of pups born alive (**H**), and litter size (**I**) were determined in dams who received intravaginal PBS or intravaginal *E. coli* K1. Data are expressed as means ± SEM (**F** and **I**). *n* = 5 (**A–D**); *n* = 8 (**E–I**). ^∗∗^*P* < 0.01, ^∗∗∗^*P* < 0.001.

It first was assessed whether a nonpathogenic strain of *E. coli* K12 MG1655 (*E. coli* K12) was capable of ascending into the nonpregnant and embryonic day 16.5 pregnant uterine cavity after intravaginal infection. In nonpregnant mice, *E. coli* K12 traversed the cervical barrier by 6 hours and ascended into the nonpregnant uterine cavity by 18 hours, reaching the top of the uterine horns by 24 hours (Figure 1A). By 48 hours there was diminution of the bacterial signal. In the pregnant cohort, *E. coli* K12 ascended into the uterine cavity but neither caused premature delivery nor affected pup survival (Figure 1, B and D–F).

The consequence of an intravaginal infection with pathogenic *E. coli* K1 bacteria then was studied. The bacteria ascended into the top of the uterine cavity (Figure 1C) and subsequently induced delivery significantly earlier than mice receiving intravaginal PBS (mean time to delivery, 48.81 ± 3.51 versus 65.59 ± 2.22 h for *E. coli* K1 and control groups, respectively; *n* = 8; *P* < 0.001) (Figure 1G). Intravaginal administration of *E. coli* K1 also led to a significant reduction in the proportion of pups born alive compared with intravaginal PBS controls (mean proportion of live pups born, 0.64 ± 0.14 versus 0.98 ± 0.02; mean litter size, 6.8 ± 1.29 versus 2.98 ± 1.02 for *E. coli* K1 and control groups, respectively; *n* = 8; *P* < 0.01) (Figure 1, H and I).

### *E. coli* K1 Is Detected in the Uteroplacental Tissues and Fetus

**Figure 2:**
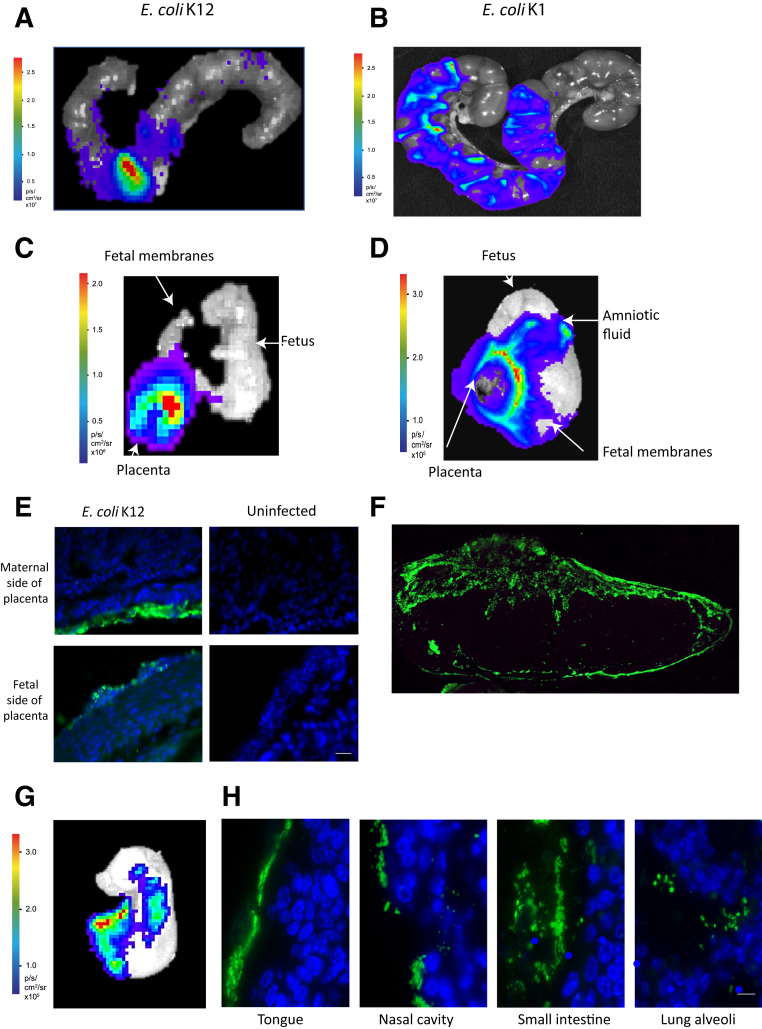
*Escherichia coli* K12 and *E. coli* K1 ascends into the uterine cavity by 18 hours after intravaginal infection. **A** and **B:** Both *E. coli* K12 (**A**) and *E. coli* K1 (**B**) are seen in the uterine cavity at 18 hours after bacterial administration. **C:** Bacteria are detectable only in the placentas of *E. coli* K12–infected dams. **D:** In *E. coli* K1–infected dams, however, bacteria are detectable in the fetal membranes, the placenta, and the amniotic fluid (fetus with intact membranes). **E** and **F:** Immunofluorescence detection of *E. coli* in placental samples derived 18 hours after intravaginal *E. coli* K12 infection (**E**) and intravaginal *E. coli* K1 infection (**F**); *E. coli* is detected on both sides of the placenta. **G** and **H:***E. coli* K1 is detected in the fetus 24 hours after intravaginal infection in the mother (**G**); immunofluorescence detection of *E. coli* in the fetus shows that bacteria are specifically present in the gastrointestinal and respiratory tracts (**H**). Scale bars: 25 μm.

After intravaginal administration of *E. coli* K12, bacteria were detected only in the placenta (Figure 2, A, C, and E). *E. coli* K1 administration led to a more diffuse spread of bacteria in the uteroplacental tissues (Figure 2B). Bacteria were detected in the fetal membranes, placenta, and amniotic fluid 18 hours after administration (Figure 2D). *E. coli* K1 and *E. coli* K12 were seen on both maternal and fetal sides of the placenta (Figure 2, E and F), with minimal bacteria detected within the central labyrinth layer (Figure 2F).

Twenty-four hours after *E. coli* K1 administration, bacteria were detected in the fetus (Figure 2G) and could be seen in the fetal respiratory and gastrointestinal tracts (Figure 2H). No bacteria were detected in the pups from *E. coli* K12–infected dams (data not shown).

### Both *E. coli* K1 and K12 Ascending Vaginal Infection Induces an Inflammatory Response in the Uteroplacental Tissues

To investigate the mechanisms behind preterm parturition after intravaginal *E. coli* K1 administration, uteroplacental inflammatory cytokine expression was compared in dams infected with *E. coli* K1 and K12 (which does not lead to premature delivery) and PBS-injected controls. Samples collected 18 hours after intravaginal administration were analyzed by quantitative PCR for inflammatory cytokine gene expression associated with preterm birth and the onset of parturition.

**Figure 3:**
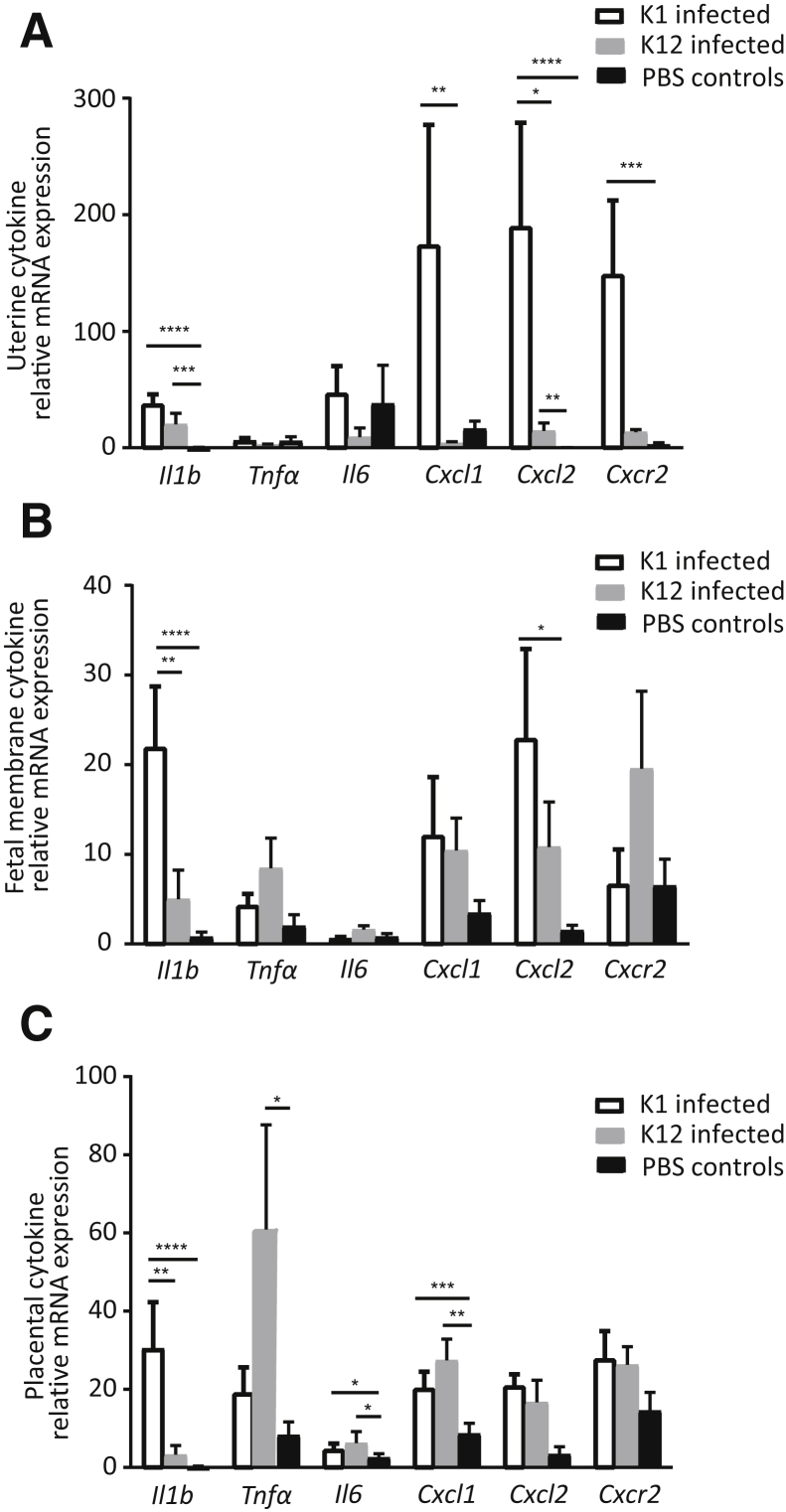
There is an up-regulation of inflammatory cytokine genes in the uteroplacental tissues after intravaginal *Escherichia coli* K1 and *E. coli* K12 administration. **A–C:** Data are expressed as mean relative mRNA expression to *GAPDH* (2^−ΔCT^) of the uteroplacental tissues in *E. coli* K1 dams, *E. coli* K12 dams, and phosphate-buffered saline (PBS) control dams. Data are not normally distributed and therefore were log transformed before analysis with a two-way analysis of variance and *post hoc* Bonferroni test. Data are expressed as means ± SEM. *n* = 3 (**A**), *n* = 6 (**B** and **C**). ^∗^*P* < 0.05, ^∗∗^*P* < 0.01, ^∗∗∗^*P* < 0.001, and ^∗∗∗∗^*P* < 0.0001.

There was significant up-regulation of *Il1b* and *Cxcl2* expression in the *E. coli* K1 and K12 uteri compared with uninfected PBS controls (K1, *P* < 0.0001 and *P* < 0.0001; K12, *P* = 0.0007 and *P* = 0.004, respectively) (Figure 3A). There also was up-regulation of *Cxcr2* expression in the *E. coli* K1 uteri compared with uninfected PBS controls (*P* = 0.0003). In the fetal membranes, there was an up-regulation of *Il1b* and *Cxcl2* expression in the *E. coli* K1 fetal membranes compared with uninfected PBS controls (*P* < 0.0001 and *P* = 0.014, respectively) (Figure 3B). In the placenta, there was up-regulation of *Il6* and *Cxcl1* expression in the *E. coli* K1 and K12 placentas compared with uninfected PBS controls (K1, *P* = 0.02 and *P* = 0.0006; K12, *P* = 0.02 and *P* = 0.0079, respectively). There was up-regulation of *Il1b* in *E. coli* K1 dams whereas there was an increase in *Tnfa* in *E. coli* K12 placentas compared with uninfected PBS controls (*P* < 0.0001 and *P* = 0.03, respectively) (Figure 3C).

Comparison of the fold changes in inflammatory cytokine gene expression between the *E. coli* K1 and K12 uteroplacental tissues showed that uterine *Cxcl1* and *Cxcl2* was increased significantly in the *E. coli* K1 dams compared with the K12 dams (32.8-fold higher for *Cxcl1*, *P* = 0.008; 16.4-fold higher for *Cxcl2*, *P* = 0.017) (Table 2). In the placenta and fetal membranes, *Il1b* was significantly higher in the *E. coli* K1 group (16.5-fold higher in the fetal membranes, *P* = 0.006; 13.9-fold higher in the placenta, *P* = 0.04).

**Table 2:** Fold Change in Gene Expression between the Uteroplacental Tissues of the *E. coli* K1 and *E. coli* K12 Dams

Gene	Uterine tissues	Fetal membranes	Placental tissue
*Il1b*	2.2	**16.5**†	**13.9**†
*Tnfa*	2.3	−2.2	−2.6
*Il6*	3.6	−2.5	2
*Cxcl1*	**32.8**†	−1.9	−1.39
*Cxcl2*	**16.4**∗	2.4	1.26
*Cxcr2*	9.6	−5.6	1.11

Data are shown as fold change in mRNA expression (2^−ΔΔCT^), analysis was performed on 2^−ΔCT^ data. Bold text denotes statistically significant results.

∗ *P* < 0.05.

† *P* < 0.01.

### Brain Inflammation Is Evident in Surviving Pups

**Figure 4:**
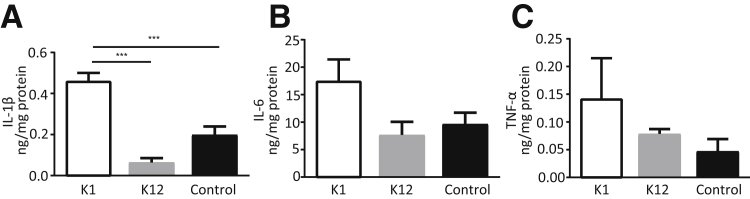
Proinflammatory cytokine IL-1β is increased in the brains of *Escherichia coli* K1–infected neonatal pups on the day of birth. **A–C:** IL-1β (**A**), IL-6 (**B**), and tumor necrosis factor (TNF)-α (**C**) protein levels from brain homogenates of postnatal day 0 pups from *E. coli* K1, *E. coli* K12, and phosphate-buffered saline (PBS) control dams (*n* = 5 to 12 from >3 dams). Data are expressed as means ± SEM. ^∗∗∗^*P* < 0.001.

To specifically assess brain inflammation, brains were collected on the day of birth to determine the protein levels of proinflammatory cytokines (Figure 4). There was a significant increase in IL-1β in the brains of pups from *E. coli* K1–infected dams compared with *E. coli* K12 pups and uninfected controls (*P* < 0.001 and *P* < 0.001, respectively) (Figure 4A), with an increased trend in IL-6 and tumor necrosis factor-α levels in the brains of *E. coli* K1 pups (Figure 4, B and C).

**Figure 5:**
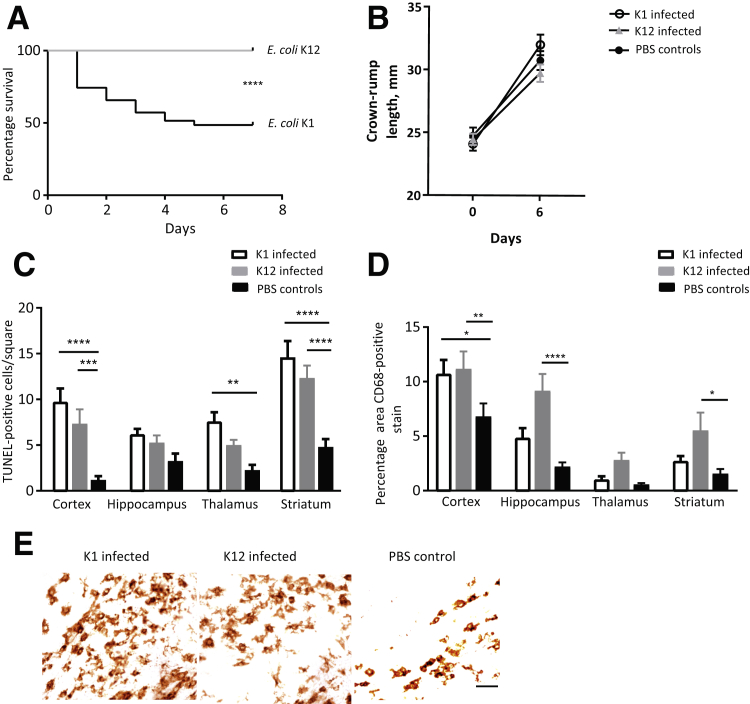
There are increased levels of CD68 and terminal deoxynucleotidyl transferase-mediated dUTP nick-end labeling (TUNEL)-positive cells in the pup brains from *Escherichia coli* K1– and *E. coli* K12–infected dams on postnatal day 6 compared with uninfected controls. **A:** Of the pups born alive from the *E. coli* K1–infected dams, approximately 50% survived to postnatal day 6 compared with 100% of pups in the *E. coli* K12 group (log-rank test *P* < 0.0001). **B:** There was no difference in the crown-rump lengths of the *E. coli* K1, *E. coli* K12, or uninfected control pups on the day of birth or on postnatal day 6. **C:** Immunohistochemical detection of TUNEL-positive cells was determined in the cortex, hippocampus, thalamus, and striatum of the pups. **D:** Immunohistochemical detection of CD68-positive cells was performed in the cortex, hippocampus, thalamus, and striatum of the pups. **E:** An example of cortical CD68 immunohistochemistry. Data are expressed as means ± SEM (**C** and **D**). *n* = 25 to 35 pups (**A**); *n* = 3 of each pup from a different dam (**E**). ^∗^*P* < 0.05, ^∗∗^*P* < 0.01, ^∗∗∗^*P* < 0.001, and ^∗∗∗∗^*P* < 0.0001. Scale bar = 25 μm. PBS, phosphate-buffered saline.

Pups born to *E. coli* K1–infected, *E. coli* K12–infected, and PBS control dams were monitored. More than 60% of pups were born alive to *E. coli* K1–infected dams (Figure 1H). Of these surviving pups, approximately 50% survived to postnatal day 6 compared with 100% of pups in the *E. coli* K12 and uninfected control groups (mean survival, 48.5% versus 100%; log-rank test *P* < 0.0001) (Figure 5A). Whole-body bioluminescent imaging of the surviving *E. coli* K1 pups showed a decline in bioluminescent signal over time (data not shown). There was no difference in the crown-rump length of the *E. coli* K1, *E. coli* K12, and uninfected control pups on the day of birth, or on postpartum day 6 (*P* = 0.74 and *P* = 0.21, respectively) (Figure 5B). To determine neuroinflammatory changes in the brains of the pups, neonatal brains were collected on postnatal day 6 for immunohistochemical analysis of CD68 (a marker for microglial cells) and TUNEL (a marker for cellular apoptosis). There was an increase in CD68-positive cells in the cortex of pups from *E. coli* K1– and K12–infected dams compared with uninfected controls (*P* = 0.01 and *P* = 0.006, respectively) (Figure 5, D and E). Interestingly, pups from *E. coli* K12–infected dams showed increased CD68-positive cells in the hippocampus and striatum (*P* < 0.0001 and *P* = 0.01, respectively). There was a significant increase in TUNEL-positive cells in the cortex and striatum in the pups from *E. coli* K1– and K12–infected dams compared with uninfected controls (cortex, *P* < 0.0001 and *P* = 0.0007; striatum, *P* < 0.0001 and *P* < 0.0001, respectively) (Figure 5C).

## Discussion

### Ascending Vaginal Infection and Preterm Birth

Here, we report the use of bioluminescent strains of *E. coli* for tracking and monitoring ascending vaginal infection and preterm birth in the pregnant mouse. Several preterm birth mouse models have been established by using local delivery of bacteria, bacterial products, or inflammatory mediators. These models have provided insight into the pathogenesis of preterm birth, for example, by identifying the importance of activator terminator-1 protein in mediating infection-related preterm birth.^26^ They have also recapitulated the process of ascending vaginal infection,^8^, ^11^, ^12^ mimicking the route of infection believed to occur in human preterm birth.^5^, ^27^

*E. coli* was selected because their associated LPS toxins are the most commonly used isolates in mouse models of inflammation-associated prematurity. In addition, *E. coli* vaginal colonization has been clinically associated with preterm birth.^28^, ^29^ The use of bioluminescent pathogens confers several advantages: the ability to track pathogens longitudinally within the same cohort of mice, which decreases the number of mice needed and increases the fidelity of the data collected; the ability to improve our understanding of how pathogens infiltrate the uterine cavity; and the ability to use bioluminescence imaging to test responses to novel therapies *in vivo* and in real time. These support two of the three Rs of animal research (replacement, reduction, refinement; *https://www.nc3rs.org.uk/the-3rs*, last accessed November 28, 2017), by reducing the number of animals required and by refining animal welfare as a result of the minimally invasive nature of the bacterial administration.

### The Impact of *E. coli* Ascending Infection on Maternal and Fetal Outcomes

These data show that *E. coli* K12 does not induce premature delivery or reduce litter size, whereas *E. coli* K1 causes preterm delivery in approximately 60% of dams within 48 hours. Clinically, the K1 strain of *E. coli* is the leading cause of neonatal sepsis and meningitis.^30^ These data are consistent with that from other preterm birth models using intravaginal live bacteria administration of the following: *E. coli* 055:B5, 50% of dams deliver within 48 hours^8^; group B *Streptococcus*, 25% to 50% of dams deliver within 72 hours.^10^, ^12^

### Bacterial Movement into the Uterine Cavity

How bacteria ascend into the uterine cavity from the vagina is a matter of debate. Bacteria may gain direct access to the amniotic cavity from a supracervical decidual region,^31^, ^32^ or may spread in the choriodecidual space before traversing the amniotic membrane.^18^, ^33^ Clinical data supporting direct amniotic cavity invasion has shown that 16S rRNA gene copy number in the amnion was significantly greater than in the chorion.^32^ In addition, in cases of chorioamnionitis, bacteria have been identified in the amniotic cavity but not in the chorioamniotic membranes. In contrast, a quarter of women with preterm chorioamnionitis showed no evidence of intraamniotic bacterial invasion or inflammation.^18^ Although the reproductive anatomy of the mouse and humans differ, bioluminescent bacteria can illuminate, practically and metaphorically, the route of bacterial vaginal infection. Here, we infer that bacteria first spread within the choriodecidual space and then to the placenta and fetal membranes. This was supported by numerous *E. coli* on the decidual side of the placenta 18 hours after *E. coli* K1 administration and within the uteroplacental tissues and amniotic fluid, before fetal colonization by 24 hours. Bacteria in the fetal gastrointestinal and respiratory tracts likely are caused by ingestion and inhalation of amniotic fluid, respectively.^34^ *E. coli* K1 A192PP-*lux2* has previously been shown to preferentially colonize the gastrointestinal tract of 2-day–old rats after oral ingestion, causing bacteremia and blood-brain barrier invasion within 48 hours.^23^ The neonatal mucus barrier of the gastrointestinal tract may not provide a sufficient barrier to invasion, increasing susceptibility to infection.^35^ This would fit with the fetal gastrointestinal colonization described in this study and the subsequent fetal outcomes seen.

### Host Response Mechanisms against Invading Bacteria

Host response mechanisms to ascending vaginal bacteria are important in preventing ascending infection and subsequent preterm birth. The pregnant cervical mucus plug is thought to limit infection by containing mucins, which sterically inhibit the diffusion of bacteria, as well as produce antimicrobial peptides.^36^, ^37^, ^38^ The amniotic cavity is also known to contain leukocytes, antimicrobial peptides, and cytokines.^39^, ^40^ Recent evidence has shown that both fetal and maternal neutrophils invade the amniotic cavity, trapping and killing bacteria.^41^, ^42^ Furthermore, mutations in innate immunity and host defense genes in neonates are associated with an increased risk of preterm rupture of membranes.^43^

### The Effect of Different *E. coli* Strains on Uterine Inflammatory Pathways and Preterm Birth

Intravaginal *E. coli* K1 leads to an up-regulation of several inflammatory genes in the uteroplacental tissues commonly associated with preterm parturition.^6^, ^7^, ^44^, ^45^ IL-1β appears to be an important mediator of labor in all of the uteroplacental tissues infected by *E. coli* K1 and this was supported by studies using IL-1β to induce premature delivery in mice.^46^, ^47^ The different concentrations of *E. coli* strains used in the pregnant dams in this study may have had differing effects on the inflammatory response. In support of this, recent data have found that bacterial DNA itself can significantly augment the inflammatory response of the host antimicrobial peptides via Toll-like receptor-9.^48^ Therefore, the ascending infection caused by these two different *E. coli* strains was not directly comparable, but can help to investigate the mechanisms of premature birth by reflecting two separate models of ascending vaginal infection: one with no effect on parturition and one resulting in preterm delivery. In the *E. coli* K12 strain, lack of the O-antigen in mature LPS renders this strain nonpathogenic, and thus less resistant to hostile environments and less likely to colonize the host and cause disease.^49^ *E. coli* is detected by the host through interaction of its LPS with the host's Toll-like receptor-4, and there is evidence that the O-antigen may interfere with Toll-like receptor-4 recognition, making the bacteria more likely to evade the host.^50^, ^51^ Different LPS serotypes can induce preterm birth in pregnant mice, and these elicit differing Toll-like receptor-4–mediated inflammatory responses supporting this hypothesis.^6^

The main differences identified in the uterine myometrial tissues were an increase in the chemokines *Cxcl1* and *Cxcl2*, and the chemokine receptor *Cxcr2* in the *E. coli* K1 group compared with the K12 group. The expression of chemokines *Cxcl1*, *Cxcl2*, *Cxcl5*, and *Cxcl8* are up-regulated in the human myometrium during labor.^52^ The cysteine-X-cysteine (CXC) chemokines, via their interaction with the neutrophil-expressed CXC receptor 1 and CXC receptor 2, mediate extravasation of neutrophils into the myometrium. This is supported by the large neutrophil influx in the decidua of *E. coli* K1 dams 24 hours after administration of intravaginal bacteria (unpublished data). Neutrophil influx into gestational tissue has been associated with term and preterm labor.^53^, ^54^ Administration of a broad-spectrum chemokine inhibitor delayed LPS-induced preterm labor by reducing neutrophil influx and LPS-induced up-regulation of IL-1β, IL-6, IL-12, CXC ligand 1, and CXC ligand 2.^55^ However, depletion of neutrophils in an intrauterine LPS preterm birth mouse model did not delay premature labor, yet reduced the local IL-1β response in the uteroplacental tissues.^56^

### Impact of Ascending Infection on Neonatal Brain Inflammation

Although some of the neurologic complications of preterm infants relate to immaturity, there is substantial evidence that perinatal exposure to infection and inflammation damages the developing brain.^6^, ^17^, ^57^, ^58^ The data in Figure 5 show that there is evidence of neuroinflammation (microglial activation and apoptosis) in brains of week-old pups after infection of both *E. coli* K1 and nonpathogenic *E. coli* K12. This is surprising because one would expect *E. coli* K1 to induce more significant neuroinflammation than K12. It is likely that the pups analyzed from the *E. coli* K1 dams represented the healthier cohort because 50% of the pups that were born alive, died within the first week of life. This suggests that in these brains, inflammation was caused by bacterial presence in the uterus, regardless of whether the bacteria were pathogenic, rather than direct systemic or central nervous system bacterial infection (because *E. coli* K12 does not infect the pup). It is known that systemic inflammation can cause rapid detrimental effects on the central nervous system before any peripheral organ dysfunction, and even in the absence of direct bacterial invasion.^59^ Placental inflammation, as mentioned previously, is a strong predictor for subsequent brain injury.^60^ Of interest, the Extremely Low Gestational Age Neonate (ELGAN) study showed that even when low-virulence microorganisms were isolated from the placenta, it could predict subsequent neonatal brain lesions and long-term diparetic cerebral palsy.^60^ The precise mechanisms of how intrauterine inflammation leads to fetal brain injury is unclear, but many animal studies have shown that, regardless of the type of pathogen used, inflammatory cytokines are likely to be the link between preterm birth and brain injury.^47^, ^61^, ^62^ Dysregulation in the normal cytokine response of the developing brain may perturb neurodevelopmental processes.^63^, ^64^, ^65^, ^66^ This may occur through transplacental passage of cytokines produced in the mother, by placental cytokines, or by cytokines produced in the fetus. IL-6, a proinflammatory cytokine expressed by leukocytes, is known to transfer freely across the placenta in an *ex vivo* human model and increased serum levels in the fetus is the hallmark of fetal inflammatory response syndrome.^67^, ^68^ Maternal IL-6 is thought to be a potential mediator of brain injury in offspring.^69^, ^70^, ^71^ Although maternal serum cytokine levels were not determined, the placentas in both *E. coli* groups had a significant up-regulation of inflammatory cytokine genes, including *Il6*. This is consistent with data from a low-dose intrauterine LPS mouse model that was insufficient to cause preterm birth but showed an up-regulation in placental inflammatory cytokines and subsequent fetal brain injury.^17^

Activated microglia in the brains of both *E. coli* K1– and *E. coli* K12–exposed pups show an increased trend of expression in all four brain regions for both strains, although they were increased significantly only in the cortex of the *E. coli* K1 pups and the hippocampus and striatum of the *E. coli* K12 pups. There was evidence of cellular apoptosis in all regions of the brain assessed in *E. coli* K1– and K12–infected brains. This is consistent with increased apoptosis in the cortex and periventricular regions of neonatal rats exposed to intracervical LPS *in utero*.^61^ Inflammation-associated brain injury may be caused by a combination of direct injury of oligodendrocytes and neurons from proinflammatory cytokines and indirect injury as a result of microglial activation by proinflammatory cytokines (e.g., Il-1β has been shown to activate hippocampal microglia in rats *ex vivo*).^72^, ^73^, ^74^ Activated microglia confer injury by further release of proinflammatory cytokines and excitatory metabolites such as glutamate, which can be cytotoxic, or by the release of oxidative free radicals.^75^, ^76^, ^77^ Microglial activation has been found to be associated with autism and is thought to contribute to the high incidence of autistic spectrum disorder among premature children.^77^, ^78^, ^79^ A limitation of this study was that only two markers of neuroinflammation were investigated in the neonatal brains. In addition to this, TUNEL staining, a common and widely used technique for detecting apoptotic cells, cannot always distinguish apoptotic from necrotic cells, and it also can falsely identify cells in the process of DNA repair. Future studies should assess other apoptosis markers, such as caspase 3 activity, as well as other histochemical markers of neuroinflammation.

## Conclusions

This study described how different strains of bioluminescent *E. coli* ascend into the pregnant uterine cavity of mice and affect the parturition process. It also begins to ascribe the specific mechanisms by which exposure to inflammation and infection within the uterus results in neurologic inflammation in the neonates. Furthermore, bioluminescence imaging of ascending vaginal infection in real time can show dynamic colonization patterns and provide new insight into infection-related preterm birth. Because the vaginal microbial environment appears to be associated so closely with preterm birth outcomes,^80^ it is critical that we improve our understanding of the mechanisms of ascending vaginal infection and its implications for fetal development. Furthermore, developing a system for tracking and monitoring these types of infection enables us to test novel therapies that can target the vagina or cervix, and to prevent these infections from occurring in the first place.

## Supplemental Data

Data Profile
